# Disrupted Rhythmicity and Vegetative Functions Relate to PTSD and Gender in Earthquake Survivors

**DOI:** 10.3389/fpsyt.2020.492006

**Published:** 2020-11-16

**Authors:** Claudia Carmassi, Valerio Dell'Oste, Carlo Antonio Bertelloni, Claudia Foghi, Elisa Diadema, Federico Mucci, Gabriele Massimetti, Alessandro Rossi, Liliana Dell'Osso

**Affiliations:** ^1^Department of Clinical and Experimental Medicine, University of Pisa, Pisa, Italy; ^2^Department of Biotechnology Chemistry and Pharmacy, University of Siena, Siena, Italy; ^3^Department of Applied Clinical Sciences and Biotechnology, University of L'Aquila, L'Aquila, Italy

**Keywords:** rhythmicity, post-traumatic stress disorder (PTSD), eating behavior, sexual function, somatic symptoms, sleep, natural disaster, vegetative functions

## Abstract

**Background:** Increasing evidence indicates that survivors to traumatic events may show disruption of sleep pattern, eating and sexual behaviors, and somatic symptoms suggestive of alterations of biorhythmicity and vegetative functions. Therefore, the aim of this study was to investigate these possible alterations in a sample of survivors in the aftermath of earthquake exposure, with particular attention to gender differences and impact of post-traumatic stress disorder (PTSD).

**Methods:** High school senior students, who had been exposed to the 2009 L'Aquila earthquake, were enrolled 21 months after the traumatic event and evaluated by the Trauma and Loss Spectrum Self-Report to investigate PTSD rates and by a domain of the Mood Spectrum Self-Report–Lifetime Version (MOODS-SR), to explore alterations in circadian/seasonal rhythms and vegetative functions.

**Results:** The rates of endorsement of MOODS-SR *rhythmicity and vegetative functions* domain and subdomain scores were significantly higher in survivors with PTSD with respect to those without it. Among all earthquake survivors, women reported higher scores than men on the *rhythmicity and vegetative functions* domain and subdomain scores, except for the *rhythmicity* and *sexual functions* ones. Female survivors without PTSD showed significantly higher scores than men in the *rhythmicity and vegetative functions* total scores and the *sleep* and *weight and appetite* subdomains. Potentially traumatic events burden predicted *rhythmicity and vegetative* functions impairment, with a moderation effect of re-experiencing symptoms.

**Conclusions:** We report impairments in rhythmicity, sleep, eating, and sexual and somatic health in survivors to a massive earthquake, particularly among subjects with PTSD and higher re-experiencing symptoms, with specific gender-related differences. Evaluating symptoms of impaired rhythmicity and vegetative functions seems essential for a more accurate assessment and clinical management of survivors to a mass trauma.

## Introduction

Post-traumatic stress disorder (PTSD) is a complex syndrome that may occur after exposure to traumatic events, characterized by severe and often chronic psychological, physiological, and cognitive symptoms. Neurobiological dysfunctions and hormonal alterations have been reported to contribute to the manifestations of the disorder, particularly to the impairment in emotional regulation, memory, and learning. Generally, these symptoms have been explored through the investigation of endocrine rhythmicity and temporal synchrony in brain activity (1). Although most of the research focused on sleep organization, studies on endocrine rhythmicity revealed that some abnormalities of both cortisol and sympathetic nervous system activity are frequently, albeit not constantly, observed in PTSD patients (2, 3). The most typical changes are a flattening of the diurnal secretion of cortisol and the hyperactivity of the sympathetic nervous system; these alterations have also been linked to an impairment in cognitive functioning, in particular in consolidation of emotional memories, attention, learning, vigilance, and arousal (4, 5).

Although there is evidence of a relationship between trauma exposure, PTSD, and sleep alterations, scant data are still available about the onset of other types of alterations in vegetative functions in survivors to traumatic events, such as eating behaviors or sexual habits. Possible gender differences in such symptomatology have been even reported to a lesser extent. Current literature recognizes that women may have a two-fold risk for PTSD, with respect to men exposed to the same traumatic event (6–9). Again, increased evidence suggests the former to report more severe symptoms than the latter, with the only exception of reckless and self-destructive behaviors (10–15), although a few studies have specifically focused on neurovegetative alterations in PTSD.

Some greater short- and long-term sleep impairments have been reported in PTSD patients in the aftermath of both natural or war disasters, compared with the general population (16). These include difficulty falling asleep, engaging in atypical sleep disruptive behaviors, frequent awakenings, and nightmares (17–19). Women have also been reported to present a higher prevalence of event-related insomnia and nightmares than men (20–22).

The presence of PTSD has also been recently associated with impaired eating behaviors, such as night eating, food addiction, binge eating, maladaptive eating as a coping strategy, weight change, and overweight/obesity over time, up to the onset of full-blown eating disorders (23–25). Moreover, in samples exposed to a traumatic event, significantly higher rates of eating disorders in women than in men were described (26–28).

To a lesser extent, somatic symptoms and sexual dysfunctions have also been associated with PTSD (29–32). About 50–80% of PTSD patients complain of chronic physical symptoms (33, 34). A first study carried out in sample of 142 civilian war survivors showed women to report significantly higher levels of somatic symptoms than men, whereas levels of PTSD symptoms were similar in the two sexes (35). More recently, sexual dysfunctions, such as lack of sexual desire/pleasure or pain/problems during intercourse, have been reported in subjects exposed to trauma (36–39), but limited data are available for PTSD civilian patients. In a literature revision, Yehuda et al. (36) highlighted the comorbidity between sexual dysfunctions and PTSD in war veterans and proposed some biological and psychological underpinnings of this phenomenon. Another review including 11 articles on sexual dysfunctions in PTSD veterans showed that all but one study reported a significantly increased prevalence of sexual dysfunctions, especially erectile ones and decreased sexual desire (37). Finally, a recent study on 300 veterans with PTSD showed that sexual dysfunction was predicted by the severity of the D cluster of PTSD symptoms (39).

Italy is among the most seismically active countries in Europe, although deadly earthquakes are not very common. On April 6, 2009, an earthquake of Richter Magnitude 6.3 struck L'Aquila, Italy, a town with a population of 72,000 residents and a health district of 105,000 residents. The toll of the earthquake included 309 deaths, more than 1,600 individuals injured, and 66,000 displaced.

A great part of central Italy was involved by the seismic event, and large parts of the town of L'Aquila were destroyed. The final evaluation of the damages showed this as the fifth more devastating earthquake in Italy in the last century.

Given the paucity of available information, the present study was aimed at investigating alterations in rhythmicity and neurovegetative functions, with a particular attention to the gender differences and the impact of PTSD symptoms, among survivors of the 2009 L'Aquila earthquake in Italy.

## Methods

### Participants

The target population included high school senior students living in the town of L'Aquila, who had been exposed to the 2009 earthquake, enrolled 21 months after the catastrophic event. The total sample included 512 subjects (280 men and 232 women). Other details on sociodemographic characteristics and clinical of the study sample were reported elsewhere (8, 40). The Ethics Committee of the University of L'Aquila and the school councils approved all recruitment and assessment procedures. Subjects provided written informed consent after receiving a complete description of the study, in accordance with the Declaration of Helsinki.

### Instruments and Assessments

The assessment instruments used in the present study included the modified versions of the Trauma and Loss Spectrum Self-Report (TALS-SR) (41) and the Mood Spectrum Self-Report–Lifetime Version (MOODS-SR) (42) to evaluate symptoms that occurred in the aftermath of the earthquake exposure. These instruments were developed in the framework of the international collaborative research project named *Spectrum Project*, aimed at developing and validating tools to diagnose the spectrum of clinical manifestations of the *Diagnostic and Statistical Manual of Mental Disorders* (*DSM*) disorders, and they showed a good validity and reliability (41, 42).

The TALS-SR includes 116 items exploring the lifetime experience of a range of loss and/or traumatic events and lifetime symptoms, behaviors, and personal characteristics that might represent manifestations and/or risk factors for the development of a stress response syndrome. It is composed by nine domains, namely, *loss events (I), grief reactions (II), potentially traumatic events (III), reactions to losses or upsetting events (IV), re-experiencing (V), Avoidance and numbing (VI), maladaptive coping (VII), arousal (VIII)*, and *personal characteristics/risk factors (IX)*. According to previous studies also including populations of young adults (40, 43, 44), *DSM-5* PTSD diagnosis was assessed utilizing the following matching between symptom criteria and TALS-SR items: criterion B (B1 = 80, B2 = 77, B3 = 79, B4 = 78, and B5 = 81), criterion C (C1 = 86, C2 = 87 and/or 88 and/or 89), criterion D (D1 = 90, D2 = 95, D3 = 85, D4 = 96, D5 = 91, D6 = 93, and D7 = 92), and criterion E (E1 = 108, E2 = 99 and/or 100 and/or 102 and/or 103 and/or 104, E3 = 106, E4 = 107, E5 = 105, and E6 = 109). Because of the sample characteristics, criterion A was considered satisfied.

Trauma and Loss Spectrum Self-Report presented good intraclass correlation coefficients (from 0.934 to 0.994) with SCI-TALS, the interview version used for assessing post-traumatic stress symptomatology. SCI-TALS, similarly, showed a good internal consistency (Kuder–Richardson coefficient exceeding the minimum standard of 0.50 for each domain) (41).

The MOODS-SR is a 140-item questionnaire exploring mood spectrum symptoms, coded dichotomously, as present or absent, for one or more periods of at least 3–5 days. According to previous researches and to the aims of the present study (11, 26, 43), we adopted a modified version of the instrument assessing symptoms developed in the aftermath of earthquake. Items are organized into three manic and three depressive domains, exploring “mood,” “energy,” and “cognition,” besides a *rhythmicity and vegetative functions* domain. This latter explores alterations in the circadian rhythms and vegetative functions, including changes in energy; physical well-being; mental and physical efficiency related to the weather and season; and changes in appetite, sleep, and sexual activities across 29 items (26, 43).

The *rhythmicity* subdomain consists of six items investigating the changes in mood, energy, interests and efficiency during the course of the year or even during the day, according to the weather, the season, and the phase of menstrual cycle or in case of disruption of circadian rhythms. The vegetative functions subdomains are *sleep* (12 items, investigating insomnia, sleepiness, reduced need for sleep, changes in sleep related to external stimuli, season, jet-lag syndrome, and menstrual cycle), *weight and appetite* (4 items, concerning changes in taste, changes in appetite and weight, craving for carbohydrates), *sexual functions* (5 items, examining the reduction of sexual interest, difficulties with sexual stimulation and orgasm, increased sexual interest, and tendency to promiscuity), and *physical symptoms* (5 items, regarding headache, xerostomia, constipation, nausea, and other gastrointestinal problems).

Mood Spectrum Self-Report–Lifetime Version, the self-report version of SCI-MOODS, presented good intraclass correlation coefficients (from 0.88 to 0.97) with the interview format (SCI-MOODS).

SCI-MOODS, the interview version for assessing mood symptomatology, had a good internal consistency (Cronbach α ranged between 0.72 and 0.92) (42).

### Statistical Analysis

The Mann–Whitney *U* tests were computed in order to compare MOODS-SR domain scores and vegetative functions subdomain scores in men vs. women, and PTSD vs. non-PTSD survivors.

Furthermore, to study the possible interaction effects of gender and PTSD on each MOODS-SR domain and *rhythmicity and vegetative functions* subdomain score, we performed the gender comparison within the subsample of patient with and without PTSD.

A multiple linear regression was used to study the relationships between the TALS-SR domains and the MOODS-SR *rhythmicity and vegetative functions* domain and to identify the strongest predictors. Subsequently, taking into account the TALS-SR domains that showed a significant association with the MOODS-SR *rhythmicity and vegetative functions* domain, a moderation analysis (with predictor and moderator centered before the analysis) was conducted. The Hayes's PROCESS tool was utilized.

All statistical analyses were carried out using the Statistical Package for Social Science (SPSS Inc., Chicago, IL, 2018), version 25.0.

## Results

Full data were available for 450 subjects, of whom 197 (47.8%) were women and 253 (56.2%) were men (mean age ± SD, 17.64 ± 0.78 years). A PTSD diagnosis, according to the *DSM-5* criteria, was present in 162 (36.0%) subjects, specifically in 61 men (24.1%) and 101 women (51.3%).

The rates of endorsement of the MOODS-SR *rhythmicity and vegetative functions* domain were significantly higher (*p* < 0.001) among survivors with PTSD (2.81 ± 3.67) with respect to those without it (0.98 ± 2.67). Statistically significantly higher total domain scores were present in the total sample in female (1.95 ± 2.93) than in male survivors (1.40 ± 2.99) (*p* < 0.001). Again, women without PTSD (1.18 ± 2.06) showed higher scores than men (0.88 ± 2.33) (*p* = 0.008).

All the MOODS-SR *rhythmicity and vegetative functions* subdomain scores were significantly higher in survivors with PTSD than in those without it (Table 1).

**Table 1:** Comparison of MOODS-SR *rhythmicity and vegetative functions* subdomain scores among L'Aquila earthquake survivors with (*n* = 162) and without (*n* = 288) PTSD.

***Rhythmicity and vegetative*** ***functions* subdomain scores**	**No PTSD**	**PTSD**	
	**Mean ± SD**	**Mean ± SD**	***p***
Rhythmicity	0.14 ± 0.56	0.45 ± 1.00	<0.001
Sleep	0.50 ± 1.34	1.30 ± 1.92	<0.001
Weight and appetite	0.11 ± 0.39	0.45 ± 0.81	<0.001
Sexual functions	0.13 ± 0.47	0.30 ± 67	<0.001
Physical symptoms	0.11 ± 0.42	0.38 ± 0.65	<0.001

Among all earthquake survivors, statistically significantly higher mean scores of all the *rhythmicity and vegetative functions* subdomain scores, with the only exception of the *rhythmicity* and *sexual functions* subdomains, were detected in women than in men. In the subgroup of survivors without PTSD, female survivors showed significantly higher scores in the *sleep* and *weight and appetite* subdomains with respect with male ones (Table 2).

**Table 2:** Gender differences in MOODS-SR *Rhythmicity and vegetative functions* subdomain scores in the total sample (*n* = 450) and in L'Aquila earthquake survivors with (*n* = 162) or without (*n* = 288) PTSD.

***Rhythmicity and vegetative functions* subdomain scores**	**Gender**	**Total sample (mean ± SD)**	***p***	**No PTSD (mean ± SD)**	***p*^**′**^**	**PTSD (mean ± SD)**	***p***″****
Rhythmicity	Male	0.25 ± 0.76	0.900	0.15 ± 0.58	0.907	0.59 ± 1.10	0.051
	Female	0.25 ± 0.77		0.14 ± 0.52		0.37 ± 0.94	
Sleep	Male	0.68 ± 1.71	0.001	0.47 ± 1.45	0.048	1.33 ± 2.22	0.518
	Female	0.92 ± 1.50		0.55 ± 1.08		1.28 ± 1.73	
Weight and appetite	Male	0.12 ± 0.40	<0.001	0.05 ± 0.25	<0.001	0.33 ± 0.65	0.183
	Female	0.39 ± 0.76		0.24 ± 0.56		0.52 ± 0.89	
Sexual functions	Male	0.21 ± 0.63	0.810	0.14 ± 0.50	0.875	0.46 ± 0.87	0.089
	Female	0.16 ± 0.44		0.11 ± 0.41		0.20 ± 0.47	
Physical symptoms	Male	0.16 ± 0.50	0.006	0.09 ± 0.38	0.212	0.38 ± 0.71	0.673
	Female	0.26 ± 0.56		0.15 ± 0.48		0.38 ± 0.61	

The multiple linear regression model, which provided the TALS-SR domain scores as independent variables and the MOODS-SR *rhythmicity and vegetative functions* domain score as a dependent variable, identified TALS-SR *potentially traumatic events (III), re-experiencing (V)*, and *maladaptive coping (VII)* domain scores as significant predictors (Table 3).

**Table 3:** Multiple linear regression: TALS-SR domain scores as predictive variables and MOODS-SR *rhythmicity and vegetative functions* domain score as dependent variable.

**TALS-SR domain scores**	***b* (SE)**	**CI_**95% min**_**	**CI_**95% max**_**	***p***
Loss events (I)	−0.04 (0.09)	−0.219	0.149	0.708
Grief reactions (II)	0.06 (0.03)	−0.007	0.117	0.081
Potentially traumatic events (III)	0.13 (0.06)	0.003	0.253	0.045
Reactions to losses or upsetting events (IV)	−0.01 (0.05)	−0.120	0.094	0.812
Re-experiencing (V)	0.19 (0.09)	0.016	0.350	0.032
Avoidance and numbing (VI)	0.10 (0.07)	−0.048	0.236	0.194
Maladaptive behaviors (VII)	0.34 (0.12)	0.106	0.582	0.005
Arousal (VIII)	0.02 (0.11)	−0.197	0.244	0.832
Personal characteristics/risk factors (IX)	0.11 (0.09)	−0.058	0.281	0.197
*k*	−0.63 (0.37)	−1.351	0.099	0.090

*R^2^ = 0.188, adjusted R^2^ = 0.171*.

Investigating on the possible interactions of TALS-SR *potentially traumatic events* with *re-experiencing* and/or with *maladaptive coping* domains, a significant moderation effect of *re-experiencing* was found. The interaction was highly significant, *b* = 0.10, CI_95%_ (0.052, 0.140), *p* < 0.001, indicating that the relationship between TALS-SR *potentially traumatic events* domain and the MOODS-SR *rhythmicity and vegetative functions* domain was moderated by the TALS-SR *re-experiencing* domain (Table 4A). Particularly, observing the conditional effect of the TALS-SR *potentially traumatic events* on MOODS-SR *rhythmicity and vegetative functions* domain at the values of the moderator TALS-SR *re-experiencing* domain, the relationship between *potentially traumatic events* and *rhythmicity and vegetative functions* domains emerged in subjects with medium–high levels of *re-experiencing* only (Table 4B).

**Table 4A:** Moderation analysis: model summary.

**Model**	***b* (SE)**	***T***	**CI_**95% min**_**	**CI_**95% max**_**	***p***
Potentially traumatic events	0.21 (0.06)	3.68	0.097	0.318	<0.001
Re-experiencing	0.35 (0.06)	5.94	0.231	0.459	<0.001
Potentially traumatic events **×** Re-experiencing	0.10 (0.02)	4.29	0.052	0.140	<0.001
*k*	1.50 (0.13)	11.32	1.235	1.754	<0.001

*R = 0.420, R^2^ = 0.176*.

**Table 4B:** Conditional effects of the focal predictor at values of the moderator(s).

**TALS-SR** **Re-experiencing domain**	**Effect *b* (SE)**	***t***	**CI_**95% min**_**	**CI_**95% max**_**	***p***
−2.29	−0.01 (0.08)	−0.16	−0.173	0.147	0.877
0.00	0.21 (0.06)	3.68	0.097	0.318	<0.001
2.29	0.43 (0.07)	6.06	0.289	0.567	<0.001

## Discussion

To the best of our knowledge, this is the first study to specifically explore alterations in rhythmicity and vegetative functions in young adults surviving a massive earthquake, with a particular attention to the association with gender and PTSD diagnosis.

As previously reported in a related study (8), females reported double rates of PTSD diagnosis with respect to males. This result is in line with previous findings from other researches, in the aftermath of a disaster, in which females were twice as likely to develop a full PTSD rather than males, with mean rates up to more than 50% (45–47).

Our data highlighted the presence of a sort of disruption in rhythmicity and vegetative functions, with statistically significantly higher rates in survivors with than in those without PTSD. Even if we have no follow-up data on the same cohort of subjects, we hypothesize that the PTSD symptomatology, emerged in our sample, had a chronic course, with a stability of symptoms over time, as shown by previous studies on similar samples in the aftermath of a catastrophic traumatic event (8, 10, 26, 40, 48, 49). So, we may argue that alterations in rhythmicity and vegetative functions, specifically emerged in our research in the aftermath of the earthquake, were related to the PTSD symptomatology.

Despite female survivors showed significantly more sleep alterations, impairment in eating behaviors, and somatic symptoms than male ones, a trend toward a similar symptom severity across gender in survivors with PTSD can be observed. In particular, comparing survivors with and without PTSD, we found significant differences in the *rhythmicity* subdomain. Previous studies described how alterations in the rhythmicity of endocrine secretion, sleep patterns, or temporal synchrony of brain activity could be related to PTSD severity (1). Noteworthy, no gender differences emerged in *rhythmicity* symptoms in our sample, while suggesting a similar pattern of dysregulation across genders that should be further investigated in larger samples. It is interesting to highlight that alterations were actually slightly higher in men with PTSD. We may argue that these disturbances in circadian rhythms could be related to a mood spectrum diathesis in men already found to be associated with a higher incidence of maladaptive behaviors (10–13).

According to the current diagnostic criteria, sleep alterations are considered a core symptom of PTSD (50, 51). Approximately 70% of PTSD individuals report co-occurring sleep problems, such as greater trouble initiating and maintaining sleep (19), which might contribute to the development and maintenance of the disorder (52–54). Indeed, sleep problems prior to any traumatic event may increase the likelihood of developing PTSD (55), and they often do not remit after PTSD-focused interventions (56). Recently, sleep alterations were also identified as potential risk factors for suicidal ideation and attempts in PTSD (57, 58). Our results corroborated the higher prevalence of these symptoms in women exposed to a traumatic event, even without the onset of a full-blown post-traumatic symptomatology (20–22). Kobayashi et al. (21), in a sample of 45 (17 women and 28 men) non-amnesic injured patients admitted to a trauma center, reported women to present more nightmares and sleep-interfering disruptive nocturnal behaviors, especially hot flashes and memories/nightmares of trauma soon after their injuries, compared with men. Ansara and Hindin (20) collected data from a sample of 676 female and 455 male victims of interpersonal violence and highlighted a greater presence of sleep problems in the former than in the latter exposed to similar traumatic experiences. More recently, after examining the prevalence of insomnia and nightmares within a national sample of 2,647 adults who had been exposed to one or more potentially traumatic event, women were more likely to endorse experience of insomnia and nightmares compared to men, while individuals suffering from PTSD showed no gender-related differences (22).

Association between traumatic life events and dysfunctional eating pattern has also been widely studied (59–62). Findings suggest that emotional eating is common in veterans reporting PTSD symptoms that, even of any degree of severity, seem to be associated with more emotional eating (63). Moreover, a correlation was reported between PTSD symptomatology and both underweight and obese conditions in a sample of young people who experienced different traumas (64). Post-traumatic stress disorder patients suffer from more obesity and higher waist-to-hip ratio than subjects without such disorder (65, 66). Indeed, the association between PTSD and eating disorders or diet dysregulation in the female gender has been stressed. Post-traumatic stress disorder symptoms were associated with an increased frequency of binge eating, as well as unhealthy dieting behaviors (67). In a sample of 103 women with eating disorders, 23.1% of anorexia nervosa and 25.5% of bulimia nervosa patients reported a current diagnosis of PTSD, with more severe eating symptomatology in those with cumulative traumatization (68). Mason et al. (24) reported an association between PTSD symptoms and obesity only in women with respect to men in a sample of 7,438 subjects.

Recent studies highlighted the relationship between PTSD and physical symptoms (43, 69–72). Subjects with both PTSD and chronic physical symptoms report a greater severity of the clinical picture (73, 74), a worse prognosis (73, 75), and a greater disability (76–78) than those with PTSD or physical symptoms only. McAndrew et al. (79) suggested that there could be a bidirectional relationship; that is, increases in PTSD symptoms would predict later increases in physical symptoms, or increases in physical symptoms would predict later increases in PTSD symptoms. In our sample, the physical symptoms resulted to be more compromised in women than in men, but in subjects who develop PTSD, the gender difference seemed to disappear. In this regard, contrasting data have been reported in the literature. The pathway from trauma exposure to finally reduced or impaired somatic health, mediated by PTSD, seems to be more pronounced in men than in women (80). A systematic review confirmed that individuals who reported exposure to trauma, especially men, showed an increased prevalence of functional somatic syndromes and how the magnitude of the association with PTSD was significantly stronger than with trauma exposure alone (81). Conversely, in a recent study, women showed higher levels of somatization symptoms than men, whereas levels of PTSD symptoms were similar in the two sexes (35).

This study also reported sexual dysfunctions in the aftermath of an earthquake. It has also been reported in the literature that sexual dysfunctions are greater in trauma-exposed individuals with PTSD, compared with similarly exposed survivors without it, regardless of the nature of the trauma (82–86). Our data showed sexual dysfunctions mainly in men, in both the total sample of survivors and in the subsamples with or without PTSD, although these findings were not statistically significant. Scant data are available about gender differences in sexual dysfunctions in PTSD patients and mainly concern veterans. Studies on male combat-exposed veterans found that erectile dysfunction was present in 85% of veterans with PTSD vs. 22% of veterans without it, with a three-fold increased risk of sexual dysfunction induced by PTSD (83). Furthermore, 25.1% of male veterans and 12.7% of female ones presented a sexual dysfunction diagnosis and/or prescription treatment for sexual dysfunction, especially if they were suffering from PTSD (87). Male veterans with PTSD had similar levels of sexual activity, compared with those without PTSD, whereas women with PTSD were less likely to be sexually active compared to women without PTSD. Veterans with PTSD were also less likely to report sex-life satisfaction, as compared with those without PTSD (87). In other studies on war veterans, women reported more sexual problems and less sexual satisfaction than men post-deployment (88), and female veterans who had PTSD and/or depression were more likely to report painful sex and were less likely to say sex is important and less likely to be emotionally satisfied in their relationship (89).

Our results also showed the number of potentially traumatic events, related to the earthquake, as predictors of the MOODS-SR *rhythmicity and vegetative functions* impairment, positively moderated by re-experiencing symptoms. There is evidence that traumatic experiences, including disasters, lead to changes in diurnal cortisol patterns (90–92) and that a cumulative trauma exposure may progressively disrupt circadian rhythms and other vegetative functions, including sleep, eating behaviors, or somatic complaints (70, 93–99). Our results showed the relationship between the traumatic events burden and *rhythmicity and vegetative functions* impairment in subjects with medium–high levels of re-experiencing symptoms only. A possible interpretation is that re-experiencing symptoms may lead to chronic sleep disruption, which may further pre-dispose the patient to severe distress and impairment (100, 101), and traumatic event-related thoughts may represent conditioned stimuli that elicit a conditioned waking response (102). Further, daytime re-experiencing symptoms are theorized to be the result of a failure to fully elaborate, integrate, and process traumatic event–related stimuli and subsequent information (103–105), leading to prolonged rumination on the trauma and its consequences. Interestingly, a moderate, positive relationship between rumination and PTSD symptoms in trauma-exposed adults was previously reported (106), with a stronger association between rumination and intrusive re-experiencing than avoidance or hyperarousal. In this perspective, emerging literature suggests the possible role of re-experiencing and trauma's rumination in fully mediating the relationship between distress and somatic complaints (43, 94, 107).

While interpreting the results of the present study, some important limitations should be taken into account. First is the lack of information about Axis I psychiatric comorbidities, such as mood disorders, and any related treatment, which could impact rhythmicity and vegetative functions. Second is the use of self-report instruments that could be considered less accurate than the rating of the clinician. Third is the homogeneity of the study sample that included only non-clinical high school students. Fourth is the use of a domain of a self-report questionnaire not specifically validated as independent questionnaire for assessing the daily and monthly changes in rhythmicity and vegetative functions. Finally is the lack of evaluation of the impact of chronotype on the PTSD symptomatology.

In conclusion, our results highlight impairments in rhythmicity, sleep, eating, and sexual and somatic health in survivors to a massive earthquake, particularly among subjects with PTSD, with differences between the two genders. Potentially traumatic events burden predicts rhythmicity and vegetative functions impairment, with a moderation effect of re-experiencing symptoms. Evaluating symptoms of impaired rhythmicity and vegetative functions seems essential for a more accurate assessment and clinical management of survivors to mass traumas, such as earthquakes or other catastrophic events.

## Data Availability Statement

The datasets generated for this study are available on request to the corresponding author.

## Ethics Statement

The studies involving human participants were reviewed and approved by Ethics Committee of the University of L'Aquila. The patients/participants provided their written informed consent to participate in this study.

## Author Contributions

CC, AR, and LD participated to the conception and design of the study. CC and VD participated to the interpretation of the data, the draft and critical revision of this article. VD and GM undertook the statistical analysis. CC, VD, GM, AR, CF, ED, and FM participated to the critical revision of the manuscript. All authors agreed to be cited as co-authors, accepting the order of authorship, and approved the final version of manuscript and the manuscript submission to Frontiers in Psychiatry.

## Conflict of Interest

The authors declare that the research was conducted in the absence of any commercial or financial relationships that could be construed as a potential conflict of interest.
